# Waiting times for systemic cancer therapy in the United Kingdom in 2006

**DOI:** 10.1038/sj.bjc.6604529

**Published:** 2008-08-26

**Authors:** M V Williams, K J Drinkwater, A Jones, B O'Sullivan, D Tait

**Affiliations:** 1 The Royal College of Radiologists, 38 Portland Place, London, W1B 1JQ UK; 2 Oncology Centre, Box 193, Addenbrooke's Hospital, Cambridge University Hospitals NHS Trust, Hills Road, Cambridge, CB2 0QQ UK; 3 The Royal College of Physicians, 11 St Andrews Place, Regent's Park, London, NW1 4LE UK; 4 Medical Oncology Department, The Royal Free Hospital, Pond Street, London, NW3 2QG, UK; 5 Department of Clinical Oncology, The Royal Marsden NHS Foundation Trust, Downs Road, Sutton, Surrey, SM2 5PT UK

**Keywords:** National audit, systemic therapy, chemotherapy, waiting times

## Abstract

This audit was conducted to measure waiting times for systemic cancer therapy across the United Kingdom. All patients, aged 16 years or older, commencing their first course of systemic therapy between 13 November and 19 November 2006 were eligible for inclusion. Data on 936 patients from 81 hospital sources were collected. Systemic therapy is largely given in compliance with national waiting time targets. In terms of the Joint Council for Clinical Oncology (JCCO) targets, 84% of patients commence treatment within 21 days and 98% of patients complied with the Department of Health target that treatment should follow within 31 days of the decision being agreed with the patient. Only 76% complied with the Department of Health 62-day target from GP referral to first definitive treatment. However, the date of urgent referral by the GP was not submitted for most patients in our survey, leaving a sample of only 84 out of 936 patients (9% of total) suitable for this analysis. There was only a 3- to 5-day difference between the waiting times for systemic therapy for patients categorised as urgent compared with routine. Locally agreed definitions had little impact on patients' priority for treatment. This audit has established a baseline measurement of waiting times for systemic therapy across the United Kingdom. The continuing introduction of novel therapies is likely to have a significant effect on the service and we recommend that service managers model the likely impact on resource requirements. In addition, urgent treatment should be clearly defined as that required within 24 h (maximum 48 h) to avoid the risk of clinical deterioration, particularly in patients with acute leukaemia, lymphoma or germ cell tumour.

It is commonly estimated that demand for systemic therapy is increasing between 5 and 10% a year. Figure 1 shows data for a single centre obtained over 15 years. It can be seen that there was a continual increase in day case activity at 15% per year. This includes supportive treatments such as transfusion and bisphosphonate infusion. There are more limited data for chemotherapy procedures and this activity increased at 5% per year. The picture is complicated because these are data from a major cancer centre where there has been continual change in practice as chemotherapy services were developed at associated cancer units.

With the continual increase in systemic therapy activity, it might be anticipated that a mismatch with demand could arise. This might then result in the development of waiting times for treatment. An audit of 5750 women treated for breast cancer in 1997–2000 to assess the effect of government targets on the treatment of breast cancer showed that the percentage of cases treated within 5 weeks was 78% for surgery, 53% for radiotherapy and 81.2% for chemotherapy (Robinson *et al*, 2003). An audit of 342 patients treated for lung cancer showed that the median wait for surgery, radiotherapy and chemotherapy was 25, 43 and 16.5 days, respectively (Devbhandari *et al*, 2007). There are also regional data from Canada on waiting times for chemotherapy (Cancer Care Ontario, 2007; Rayson *et al*, 2007), but we are not aware of any previous UK national audits to assess this problem. A search of the Medline database using the terms ‘cancer’, ‘chemotherapy’, ‘systemic’, ‘therapy’, ‘times’ and ‘waiting’ identified no other relevant publications.

Targets for cancer treatment were set in 1993 by the Joint Collegiate Council for Oncology (JCCO) (Joint Collegiate Council for Oncology, 1993). This is a joint body between The Royal College of Radiologists and the Royal College of Physicians of London. Recommended waiting time targets from the date of first oncology consultation to the start of radiotherapy or chemotherapy were as follows:

- For urgent radiotherapy or chemotherapy
  ○ Good practice – 24 h
  ○ Maximum acceptable –48 h
- For intensive (radical) chemotherapy
  ○ Good practice – 1 week
  ○ Maximum acceptable – 3 weeks (where additional specialist staging procedures are necessary).

The JCCO targets were set on the basis of professional opinion (grade D recommendation). A search of the Medline database using the terms ‘chemotherapy’, ‘delay’, ‘systemic’ and ‘therapy’ identified a single publication showing an impact of delay on outcomes (Hershman *et al*, 2006). In a study of 5003 women aged 65 years or more with breast cancer, an interval between surgery and chemotherapy of more than 3 months was associated with increased mortality and disease-specific mortality. The authors concluded that among older patients, moderate delays in the receipt of adjuvant chemotherapy occur frequently, but long delays (>3 months) are uncommon. While early initiation of therapy is of no benefit, significant delays are associated with increased mortality. Whether this reflects the medical impact of the delay of chemotherapy or factors associated with delay is unclear, but until this is clarified, patients should be encouraged to initiate treatment without significant delay (Hershman *et al*, 2006).

Anecdotally, unplanned delays in administering chemotherapy are unusual. This is in stark contrast to the situation in radiotherapy where over the last 20 years the continuing increase in demand, not matched by a compensatory increase in treatment capacity, has resulted in inevitable waiting lists (Williams *et al*, 2007). This is confirmed by the results of audits in breast and lung cancer described above (Robinson *et al*, 2003; Devbhandari *et al*, 2007). A recent systematic review of delays in radiotherapy has concluded that there is no threshold below which delay is safe and that radiotherapy should be administered as soon as reasonably achievable (Chen *et al*, 2008). This conclusion also seems reasonable for systemic therapy but there is no evidence to support it.

In United Kingdom, the NHS Cancer Plan laid out waiting time targets that have now been fully implemented (Department of Health, 2000). These comprise the following:

- *A 31-day target*: the interval from the date on which treatment has been agreed with the patient (date of decision to treat (DDT)) to first definitive treatment (FDT) for cancer therapy should not exceed 31 days.
- *A 62-day target*: the interval from urgent GP referral to first definitive treatment should not exceed 62 days.

These Department of Health targets only apply to a subset of patients, as they must have been referred urgently by their GP and be receiving their first definitive treatment for cancer. The present audit was conducted to obtain a nationwide baseline of waiting times across the four countries of the United Kingdom for systemic therapy and to compare it with the standards set by the JCCO and the Department of Health.

## Materials and methods

In the United Kingdom, there are 203 acute NHS trusts, some of which encompass several hospital sites. It is estimated that chemotherapy is provided to patients at approximately 160 of these hospitals (James, 2006), but there is no central database. Systemic therapy for cancer can be given at any hospital, which can obtain suitably formulated drugs for oral or intravenous use and arrange for their administration in compliance with the Peer Review Cancer measures (Department of Health, 2004). In addition, there is independent sector provision.

We planned to undertake an audit of waiting times for systemic therapy for cancer across the United Kingdom. To maximise participation, members of the following organisations were asked to disseminate information about participation in the audit to heads of oncology services at hospitals offering systemic therapy:

- The Royal College of Radiologists (clinical oncology audit leads).
- The Association of Cancer Physicians.
- The Cancer Network Pharmacists Forum.
- All Cancer Networks in England, Scotland and Wales.

All patients, aged 16 years or older, commencing their first course of systemic therapy during a 1-week period between 13 November and 19 November 2006 were eligible to be included. Systemic therapy included cytotoxic chemotherapy, antibody therapy and targeted small molecule therapy, whether given orally or intravenously. Hormonal therapy was excluded. The course of treatment included in the audit might have been the patient's first definitive treatment for cancer or it could have been treatment following on from radiotherapy or surgery. Prior systemic therapy was an exclusion criterion.

Data were collected using two online data collection tools (one for hospital demographic data, the other for waiting times data) between 13 November 2006 and 31 January 2007 inclusive. The tools, which had been piloted before national rollout, were designed using Snap Survey Software (Version 8), and the data were analysed using Microsoft Office Excel 2003 and Confidence Interval Analysis (Version 2.1.2).

Appendix 1 shows the data collection tool that was designed to obtain information about the diagnosis by tumour type and the regimen administered. Waiting list status was classified by each centre according to local definitions of priority (emergency within 24 h, urgent and routine). Treatment intent was categorised into curative, which included adjuvant therapy, palliative or concurrent radiotherapy/chemotherapy. In discussing the results, the categories ‘curative’ and ‘radiotherapy/chemotherapy’ were combined into ‘radical’. For all categories a ‘don't know’ option was available. Treatment dates were also collected and a detailed specification of national guidance defining these was made available, together with the relevant web links.

As there were a number of outliers, waiting times data were described using the median and interquartile range (IQR). To compare differences in waiting times between subgroups, the Wilcoxon two-sample test was used. This is equivalent to the Mann–Whitney *U*-test.

## Results

**Figure 2:**
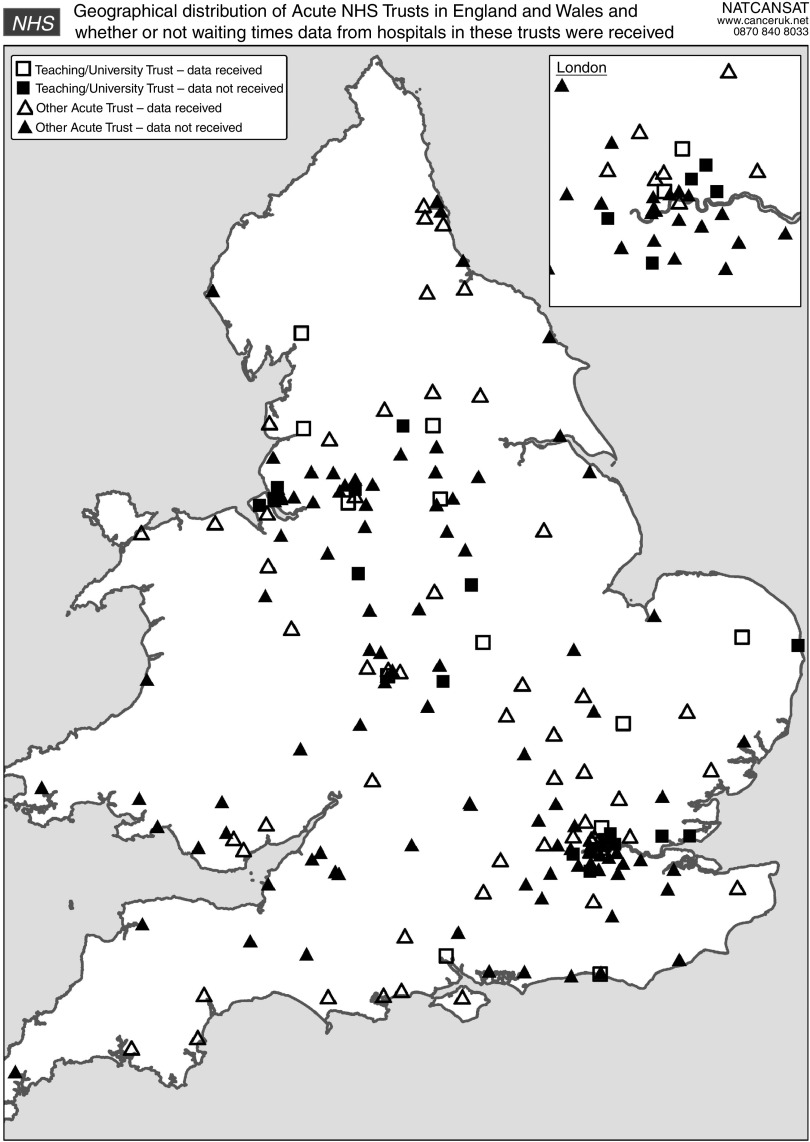
Geographical distribution of acute NHS trusts in the United Kingdom and Wales categorised by whether or not waiting times data were received.

Data were collected on 936 patients from 81 individual and combined hospital sources (e.g,, multihospital NHS trusts). In terms of the geographical distribution of the hospital sources that submitted waiting times data, 76 (94%) were in the United Kingdom or Wales, 4 (5%) were in Scotland and 1 (1%) in Northern Ireland (see Table 1). The 76 UK and Welsh hospitals were drawn from 67 out of 184 acute NHS trusts in the two countries. However, not all acute trusts offer chemotherapy. Of these, 13 (19%) trusts represented were teaching or university trusts (For the purpose of this audit, a teaching/university trust was defined by ‘teaching’ or ‘university’ in the title of the organisation.) and the proportion is similar (14% among those who did not submit data). Figure 2 shows that there does not appear to be a bias towards any particular parts of the United Kingdom and Wales with regard to participation.

**Figure 3:**
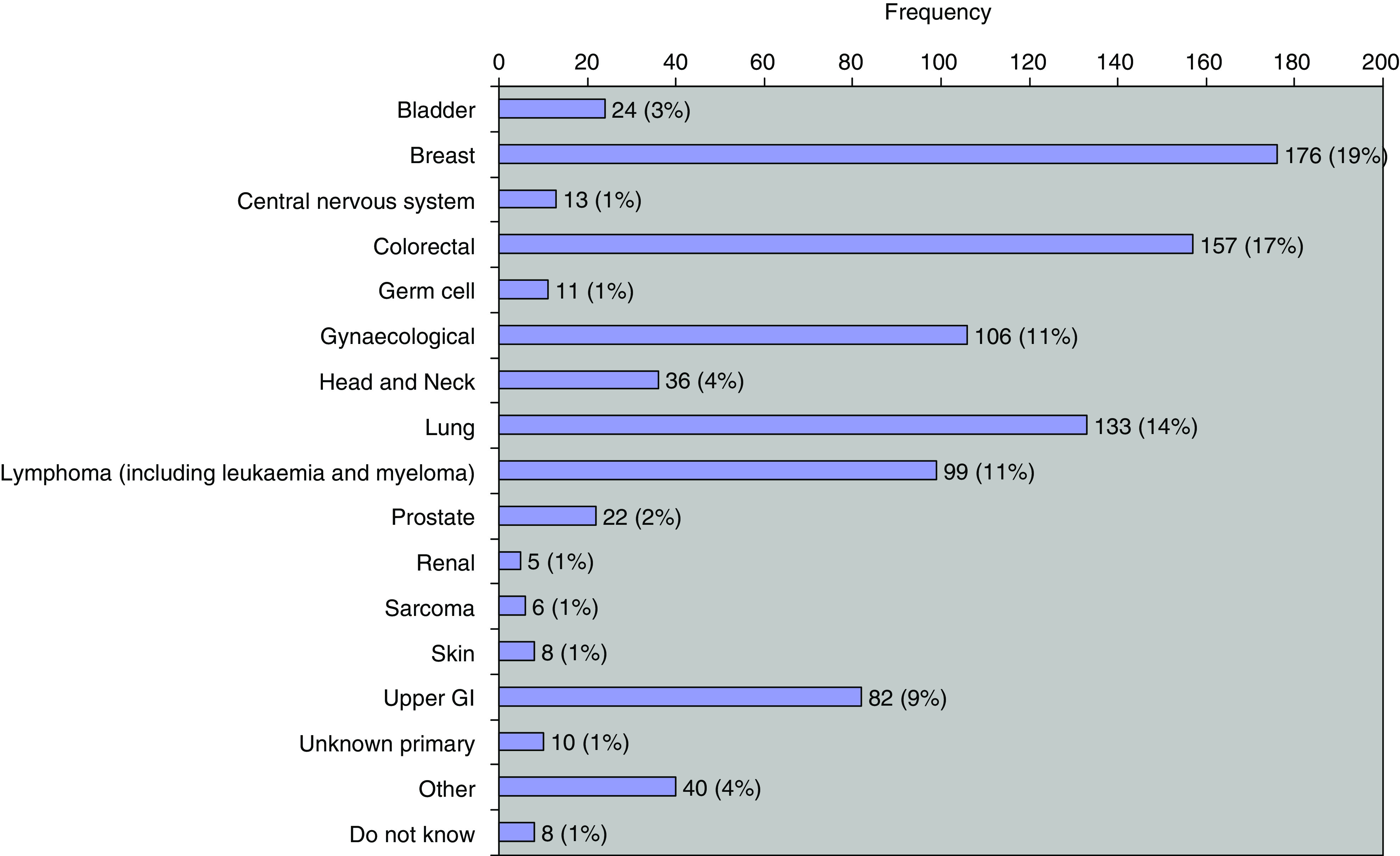
Diagnostic categories.

**Figure 4:**
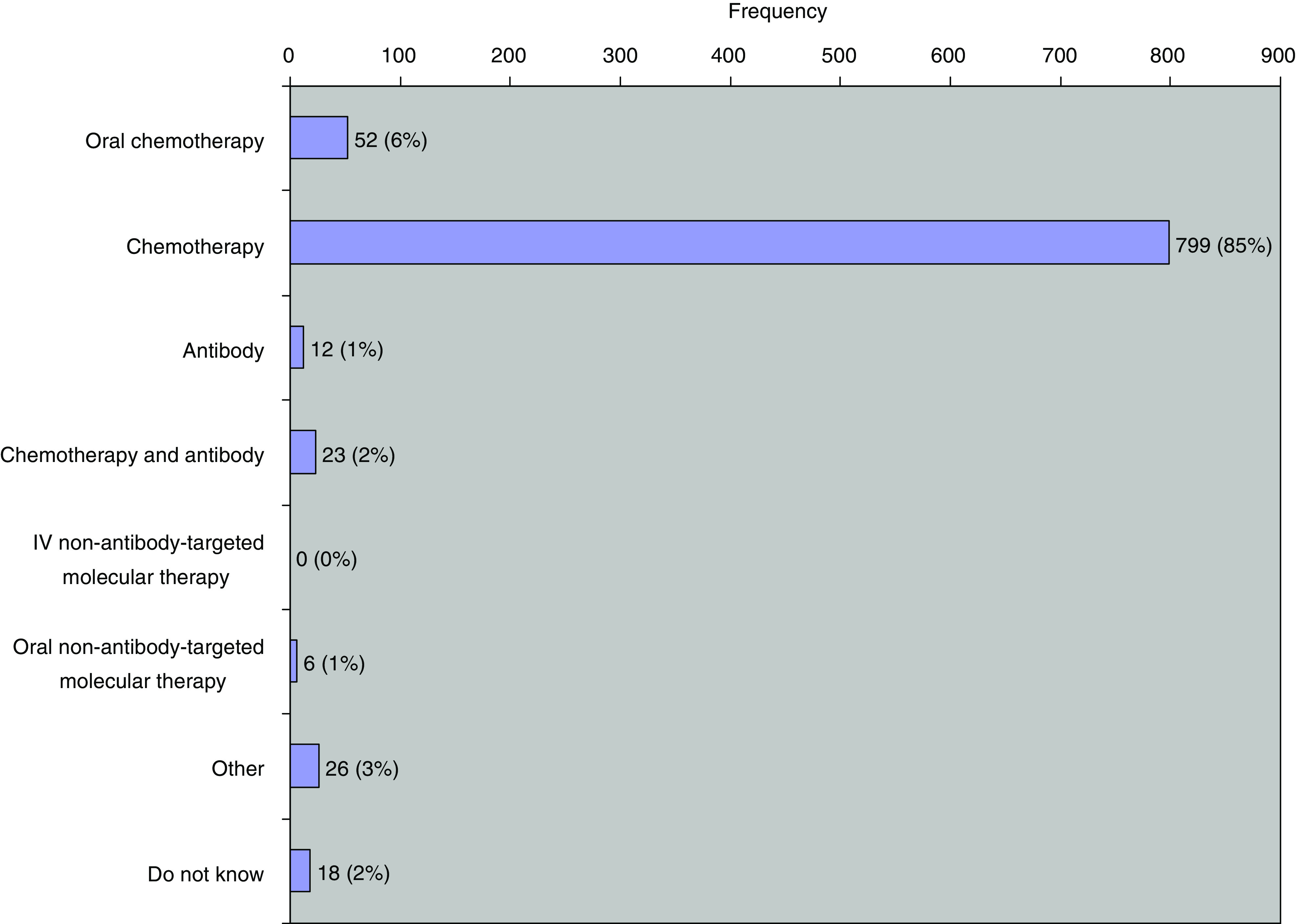
Treatment categories.

Figure 3 shows that the most frequent diagnosis was breast cancer, followed by colorectal and lung cancer. Figure 4 shows that intravenous chemotherapy comprises the most frequent category of systemic therapy at 85%. Treatment intent was curative (including adjuvant) in 38%, palliative in 49% and don't know in 8%. Chemo/radiotherapy was administered to 6%, making a total of 44% treated radically.

In terms of waiting list status, 1% of cases (8) were emergencies, 27% (252) were urgent, 48% (445) were routine and 25% (231) were unknown. Forty-one per cent (388) of treatments were FDT. Fifteen per cent (141) of all treatments were subject to elective delay. The reasons given for elective delay (140) (One of the data was missing.) were recovering from surgery (18%; 25), patient request (19%; 26), intercurrent illness (9%; 13), don't know (1%; 2) and other (53%; 74). Elective delays were excluded from further calculations.

**Figure 5:**
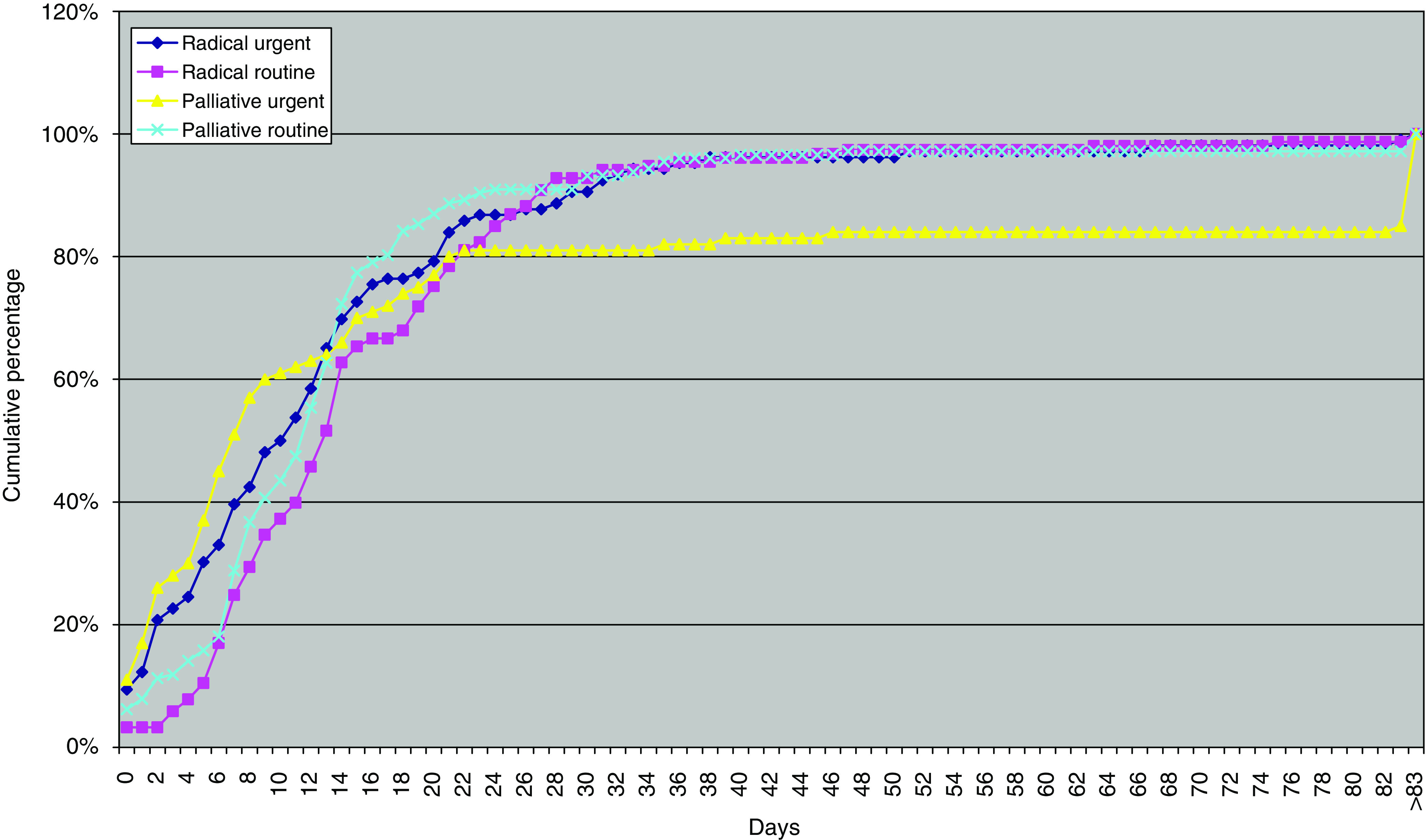
Waiting times from date treatment plan agreed with patient to first administration of systemic therapy by treatment intent and waiting list status.

Figure 5 shows waiting times for treatment categorised by treatment intent and by waiting list status. Overall, there is only a small improvement in the rapidity of treatment for those categorised as urgent rather than routine. The median waiting time from first oncology consultation to the start of urgent chemotherapy was 9 days (IQR 16 days) and from first oncology consultation to the start of routine chemotherapy was 12 days (IQR 11 days) (Figure 5A). Patients categorised as requiring urgent palliative therapy were treated fastest (Figure 5B). Waiting times by treatment intent and by waiting list status are shown in Table 2. Patients in the urgent category were treated between 3 and 5 days sooner. These differences in median waiting times were significant for overall and for radical cases, but not for palliative cases using the Wilcoxon two-sample test (Figure 5).

**Figure 6:**
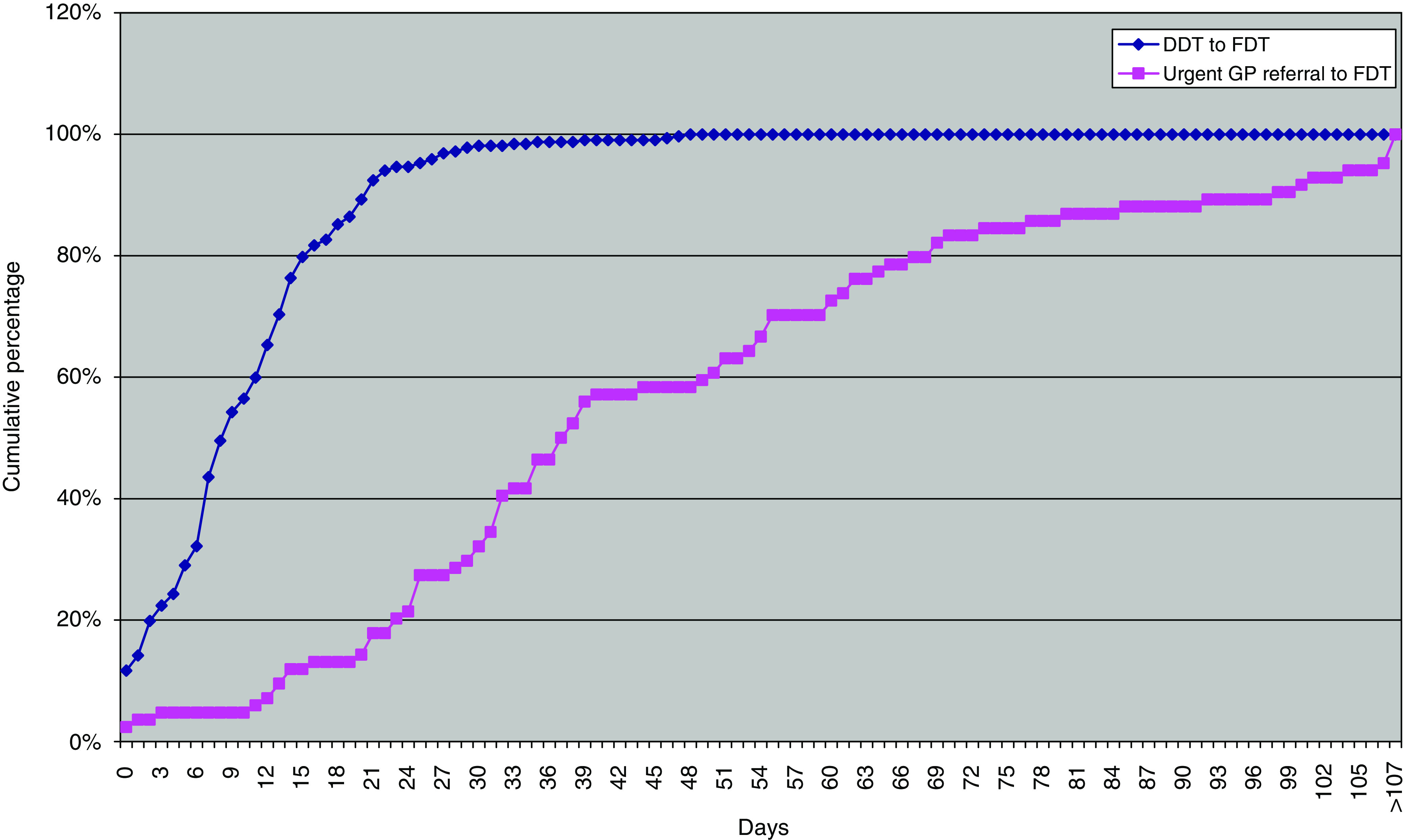
Waiting times for the Department of Health 31-day target (DDT to FDT) and 61-day target (urgent GP referral to FDT).

Figure 6 shows the results in terms of the Department of Health's 31-day and 62-day targets (Department of Health, 2000). The median time from DDT to FDT was 9 days (IQR 9 days) and from urgent GP referral to FDT 38 days (IQR 37 days). Table 2 shows compliance with the treatment targets as defined by Joint Collegiate Council for Oncology (1993) and by the Department of Health (2000). Achievement of the Department of Health 31-day target was 98.1%. Performance on the 62-day target is less satisfactory at 76.2%. The JCCO target that radical chemotherapy should start within 21 days was met in 83.7% of cases, but the recommendation that urgent treatment should start within 2 days was only met in 23.4% of cases (Table 3).

**Figure 7:**
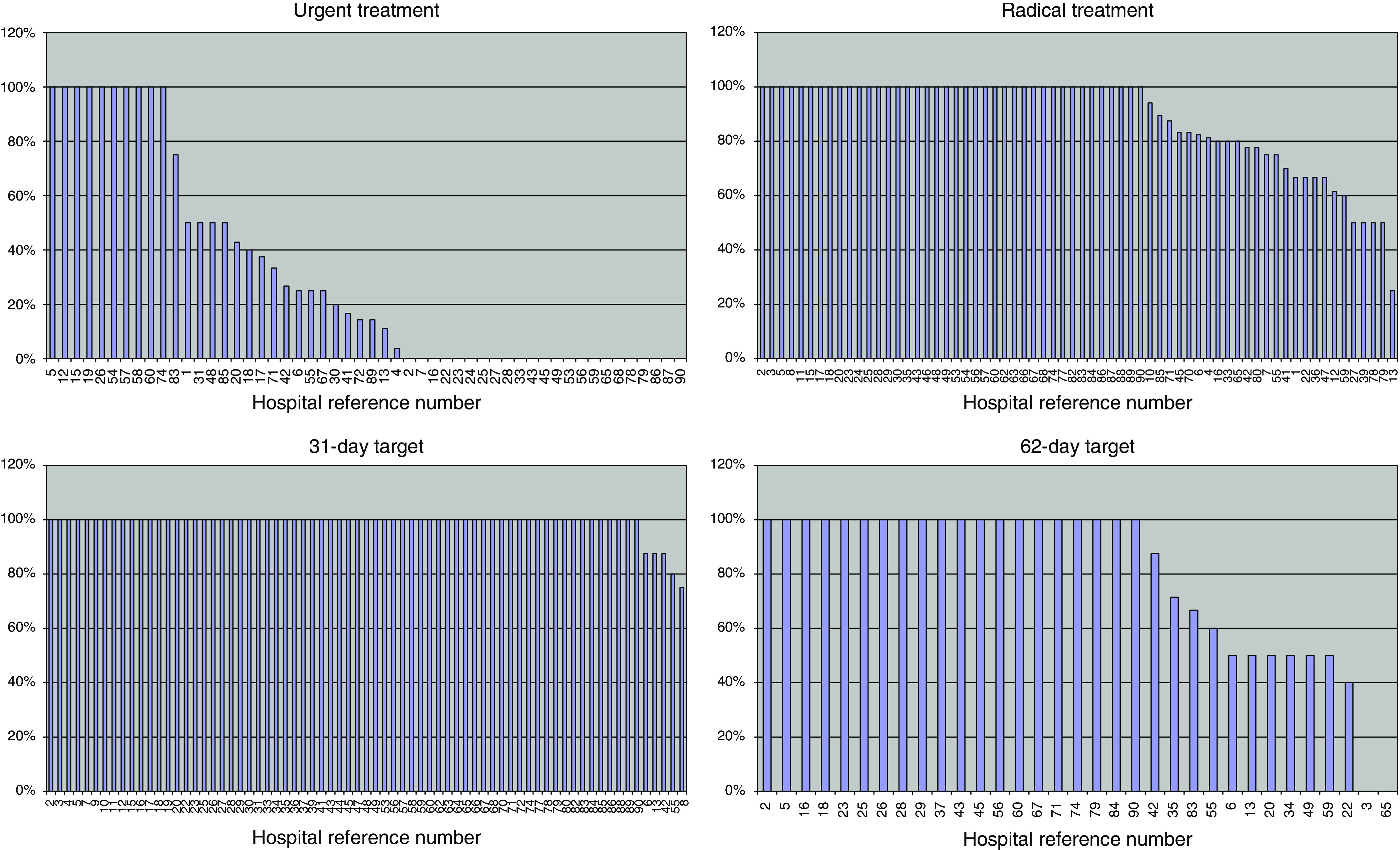
Percentage treated within target. Hospital reference numbers are shown along the *X*-axis.

Figure 7 shows the results for individual hospital units ranked in order and identified by their individual code number. The varying numbers in each chart reflect the number of patients in the different categories. For the 62-day target, there were 320 patients whose systemic therapy was their FDT and who were not subject to elective delay. However, the date of urgent referral by the GP was only available on 84 patients (26%). Lack of the appropriate data probably influenced the apparently low achievement of the 62-day target, with only 61% (20 of 33) of hospitals achieving it for all patients, compared with 93% (66/71) for the 31-day target. There was poor compliance with the JCCO target that urgent treatment should be given within 48 h, with only 10 hospitals (19%) achieving it for all their patients.

## Discussion

We report a first attempt to measure waiting times for systemic therapy for cancer across the United Kingdom. We received data from 81 individual and combined hospital sources and estimate that this comprised about half of trusts administering such treatment to patients. Every effort was made to keep non-responses to a minimum, by asking for small amounts of data, and several mechanisms were used to disseminate the data collection tools as widely as possible. The data were checked to ensure that we had not received duplicate responses from the same trust.

These data provide a snapshot of current workloads from a sample of over half of the trusts administering such treatment. These data were not population based, and it was not possible to determine whether or not case ascertainment was complete in the trusts that did submit data. However, previous national audits of radiotherapy waiting times have shown good concordance between data collected in a 1-week snapshot and large national data sets (Williams *et al*, 2007). In that survey in 2005, 1 week's data from all 57 radiotherapy centres in the United Kingdom was compared with a sample of activity for the financial year 2004/05 for 36 English centres. The estimates of annual activity agreed within 3% for patients and 6% for treatment fractions (Williams *et al*, 2007).

The cause of variable waiting times for treatment was not identified in this audit, as we did not attempt to subdivide the wait according to the intervals between the first oncology consultation, the decision to treat and treatment delivery. This might be a topic for local audit to identify where delays occur. No analysis of a possible association between long waiting times and clinical or epidemiological characteristics was attempted. As 84% of patients were treated within 21 days, any such issues could be resolved by local prioritisation.

The impact of delays will vary according to patient diagnosis; a short delay or interruption between treatment courses could be critical in the management of a rapidly proliferating leukaemia, lymphoma or germ cell tumour but would be expected to have much less impact on slower growing malignancies. In addition, we only looked at the delay in commencing the first definitive systemic therapy treatment. We did not analyse interruptions to treatment later in the course that may also be critically important for some subsets of patients. This paper presents no data on outcomes as this would require long-term follow-up of individual patients and we did not collect patient identifiable data centrally.

Figures 3 and 4 show the comparative frequency of different malignancies and of different systemic therapies. The latter are dominated by chemotherapy at 85% (Figure 4). Even within this category, there are substantial variations in administration time. Simple bolus chemotherapy may be administered in 30 min, but many drugs take longer time. For example, docetaxel takes an hour, paclitaxel takes 3 h and cisplatin takes 8 h with the associated necessary intravenous hydration. Antibody therapy shows a similar spectrum of administration time. Rituximab takes 4 h for a first administration but can be shortened for subsequent doses. Trastuzumab takes 90 min with observation for the rest of the day but subsequent infusions are given in 30 min. The length of time for which a patient is on treatment, particularly in the metastatic setting, may be very prolonged, possibly even for years, thereby adding to the long-term workload for a systemic therapy unit.

It is important to note that many of the newer targeted therapies, such as small-molecule tyrosine kinase inhibitors, are given orally and the workload for administration will therefore be low, although patient assessment and safety monitoring may be more complex. There are therefore very substantial variations in medical, nursing and couch-time resource implications for patients receiving systemic therapy, which are not fully identified in our audit.

Five-year survival of cancer in Europe continues to improve, but in the United Kingdom survival for all cancers combined remains below the European average, similar to that of some eastern European countries (Verdecchia *et al*, 2007). Late diagnosis has been highlighted as a major remediable cause of poor outcomes (Richards, 2007), but underinvestment in cancer drugs may also contribute (Anonymous, 2007). Practise will continue to change as new agents become available, and as more lines of therapy are offered to patients for a wider range of cancers and to patients who are older and of lower performance status. In addition, there are national initiatives to provide cancer services nearer to the patient's home and ambulatory care is highlighted in the Cancer Reform Strategy (Department of Health, 2007a). The issues of service planning are being addressed in the C-PORT initiative led by the Cancer Action Team at the Department of Health (Cancer Action Team, 2006). This provides a resource planning tool for departments administering systemic therapy to cancer patients.

Figure 5 and Table 2 show that there is, in practise, little distinction in the waiting time to start treatment between the JCCO categories of urgent and routine, as currently self-defined by departments across the United Kingdom. The poor compliance with the JCCO target that urgent treatment should be given within 48 h probably reflects inappropriate categorisation, as 38% of all patients were so categorised (Table 2). We suggest that urgent systemic therapy should be defined as that required within 24 h (maximum 48 h) to avoid the risk of clinical deterioration, particularly in patients with acute leukaemia, lymphoma or germ cell tumour.

Performance across the United Kingdom against the Department of Health 31-day and 62-day targets shows that there is high compliance with the 31-day target at 98.1% of patients. The result for the 62-day target is less satisfactory at 76.2%, on a sample of only 84 out of 936 patients (9% of total), and 74% of otherwise eligible cases were excluded because the date of urgent referral by the GP was not submitted in our survey. The Department of Health cancer waiting times database for the United Kingdom for the same period (quarter 4, 2006/7) includes a much larger sample of 4538 patients treated both with chemotherapy and with hormones, which were excluded from our study (Department of Health, 2007b). For the administration of these anti-cancer drugs, there was 96% compliance when only a single trust was involved and 91% compliance where inter-trust transfers were involved (Di Riley, Cancer Action Team, personal communication), for an overall result of 96% compliance (Department of Health, 2007b). Further analysis would be required to determine the cause of the discrepancy, but these results are not necessarily incompatible with each other. The strength of our study, however, was that it also included the 66% of patients who are not measured by this target.

## Conclusion

This audit has established a baseline measurement of waiting times for systemic therapy across the United Kingdom. The continuing introduction of novel therapies will have a significant effect on the demands on the service. We recommend that service managers model the likely impact on resource requirements, possibly using the C-PORT tool being developed by the Department of Health (Cancer Action Team, 2006). In addition, urgent treatment should be clearly defined as that required within 24 h (maximum 48 h) to avoid the risk of clinical deterioration, particularly in patients with acute leukaemia, lymphoma or germ cell tumour.

## Figures and Tables

**Figure 1:**
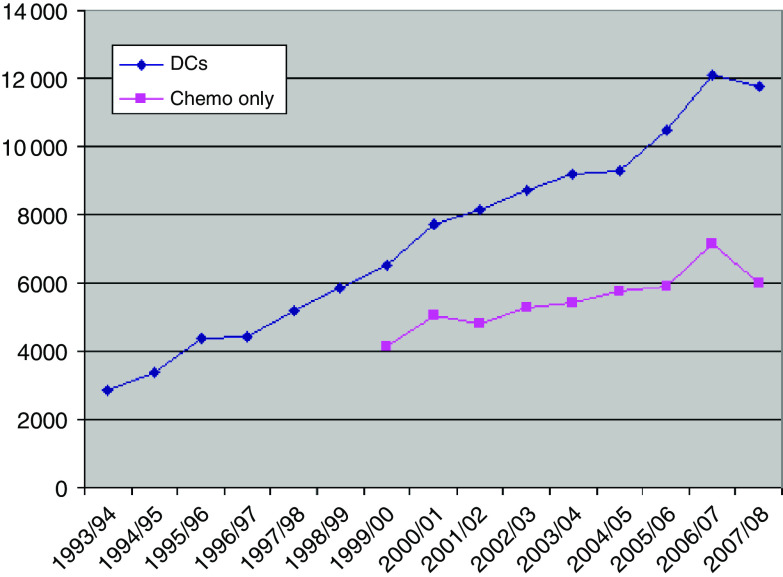
Day case and chemotherapy work load over 15 years at a large NHS teaching hospital Trust. Day case activity increased at 15% per annum and chemotherapy at 5% per annum.

**Table 1:** Response rate of trusts submitting patient data classified by country and by teaching or non-teaching status

**Country**	**Response rate % (*n/N*)**	**Response rate for teaching/university trusts % (*n/N*)**
United Kingdom	36 (61/171)	45 (13/29)
Scotland	21 (3/14)^a^	
Wales	46 (6/13)	
Northern Ireland	20 (1/5)	

a The four Scottish hospitals that responded were drawn from 3 out of 14 NHS boards.

**Table 2:** Median waiting times from date treatment plan agreed with patient to first administration of systemic therapy showing treatment intent and waiting list status

	**Waiting list status**				
	**Urgent**		**Routine**		
**Treatment intent**	**% (*n/N*)**	**M (days)**	**% (*n/N*)**	**M (days)**	***P-*value**
All^a^	38.3 (222/580)	9	61.7 (358/580)	12	0.01169
Radical^b^	40.9 (106/259)	10.5	59.1 (153/259)	13	0.00884
Palliative	36.1 (100/277)	7	63.9 (177/277)	12	0.06072

a ‘All’ comprised radical, palliative and don't know.

b Radical comprised concurrent radiotherapy/chemotherapy and curative (includes adjuvant).

**Table 3:** Compliance with targets

**Targets**	**% (*n/N*) compliance with target**	**95% CI**
1. First oncology consultation to start of urgent chemotherapy ⩽2 days	23.4 (52/222)	18.3–29.4
		
First oncology consultation to start of curative chemotherapy ⩽21 days	83.7 (273/326)	79.3–87.4
		
DDT to FDT ⩽31 days	98.1 (311/317)	96.4–99.0
		
Urgent GP referral to FDT ⩽62 days	76.2 (64/84)	67.8–83.0

The discrepancy between radical cases (259) in Table 2 and curative in Table 3 arises because the waiting list status (urgent/routine) was not recorded for 67 cases.
